# Inhibitory Effects of Respiration Inhibitors on Aflatoxin Production

**DOI:** 10.3390/toxins6041193

**Published:** 2014-03-26

**Authors:** Shohei Sakuda, Diyan Febri Prabowo, Keiko Takagi, Kazuro Shiomi, Mihoko Mori, Satoshi Ōmura, Hiromichi Nagasawa

**Affiliations:** 1 Department of Applied Biological Chemistry, The University of Tokyo, 1-1-1 Yayoi, Bunkyo-ku, Tokyo 113-8657, Japan; E-Mails: dy.prabowo@gmail.com (D.F.P.); atakagi@mail.ecc.u-tokyo.ac.jp (K.T.); anagahi@mail.ecc.u-tokyo.ac.jp (H.N.); 2 Graduate School of Infection Control Sciences, Kitasato University, Minato-ku, Tokyo 108-8641, Japan; E-Mails: shiomi@lisci.kitasato-u.ac.jp (K.S.); morim@lisci.kitasato-u.ac.jp (M.M.); 3 Kitasato Institute for Life Sciences, Kitasato University, Minato-ku, Tokyo 108-8641, Japan; E-Mail: omuras@insti.kitasato-u.ac.jp

**Keywords:** aflatoxin, fluacrypyrim, inhibitor, pyridaben, respiration, siccanin, strobirin

## Abstract

Aflatoxin production inhibitors, which do not inhibit the growth of aflatoxigenic fungi, may be used to control aflatoxin without incurring a rapid spread of resistant strains. A respiration inhibitor that inhibits aflatoxin production was identified during a screening process for natural, aflatoxin-production inhibitors. This prompted us to evaluate respiration inhibitors as potential aflatoxin control agents. The inhibitory activities of four natural inhibitors, seven synthetic miticides, and nine synthetic fungicides were evaluated on aflatoxin production in *Aspergillus parasiticus*. All of the natural inhibitors (rotenone, siccanin, aptenin A5, and antimycin A) inhibited fungal aflatoxin production with IC_50_ values around 10 µM. Among the synthetic miticides, pyridaben, fluacrypyrim, and tolfenpyrad exhibited strong inhibitory activities with IC_50_ values less than 0.2 µM, whereas cyflumetofen did not show significant inhibitory activity. Of the synthetic fungicides, boscalid, pyribencarb, azoxystrobin, pyraclostrobin, and kresoxim-methyl demonstrated strong inhibitory activities, with IC_50_ values less than 0.5 µM. Fungal growth was not significantly affected by any of the inhibitors tested at concentrations used. There was no correlation observed between the targets of respiration inhibitors (complexes I, II, and III) and their IC_50_ values for aflatoxin-production inhibitory activity. This study suggests that respiration inhibitors, including commonly used pesticides, are useful for aflatoxin control.

## 1. Introduction

Contamination of agricultural products by mycotoxins, toxic secondary metabolites of fungi, is a worldwide human and livestock health concern that has the potential for drastic economic consequences. Among the mycotoxins, aflatoxins produced by some *Aspergillus* sp. generate the most concern because of their potent toxicity, carcinogenicity [1], and high contamination in a wide variety of food and feed commodities [2,3]. Aflatoxin contamination in agricultural products is a serious problem, but it is difficult to resolve due to the lack of an effective method to control aflatoxin production.

Antifungal agents are typically applied for addressing mycotoxin contamination, however, their use can induce the rapid spread of antifungal-resistant strains [4]. A few fungicides exist that are effective against aflatoxigenic fungi in the field [5]. Additionally, specific aflatoxin-production inhibitors, which do not significantly affect fungal growth, may be useful for the control and prevention of aflatoxin contamination in food and feed without incurring a rapid spread of resistant strains. To date, some pesticides [6], microbial metabolites [7], and plant constituents [8,9] have been shown to be specific aflatoxin-production inhibitors.

We screened a natural products library (RIKEN Natural Products Depositor) and found that siccanin, a respiration inhibitor, inhibits aflatoxin production in *Aspergillus parasiticus*. Since the relationship between respiration inhibitory activity and aflatoxin-production inhibitory activity has not been reported, the aim of this study was to examine the aflatoxin-production inhibitory activity of natural and synthetic respiration inhibitors.

## 2. Results and Discussion

### 2.1. Inhibitory Activity of Natural Respiration Inhibitors on Aflatoxin Production

**Figure 1:**
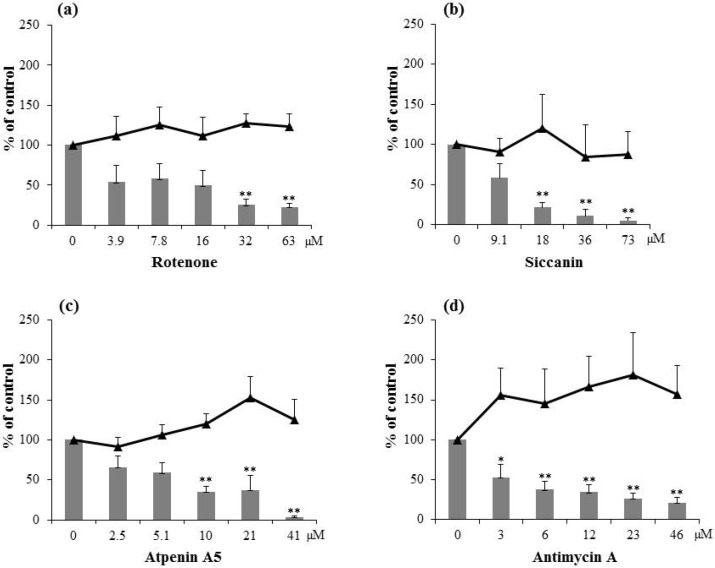
Effects of natural respiration inhibitors, rotenone (**a**); siccanin (**b**); atpenin A5 (**c**); and antimycin A (**d**), on aflatoxin (total aflatoxins B_1_ and G_1_) production (gray bars) and mycelial weight (black triangles) of *A. parasiticus*. *n* = 4–5, ** p < 0.01; * *p* < 0.05, *vs.* control.

All of the four natural respiration inhibitors with different targets (complexes I, II, and III in the mitochondrial respiratory chain) inhibited aflatoxin production of *A. parasiticus* in a dose-dependent manner (Figure 1a–d). The IC_50_ value required for each compound to inhibit aflatoxin production of *A. parasiticus* is shown in Table 1. Rotenone (a complex I inhibitor), siccanin and atpenin A5 (complex II inhibitors), and antimycin A (a complex III inhibitor) had similar activities with IC_50_ values around 10 µM. None of the four inhibitors significantly reduced fungal mycelial weight at the concentrations tested. This indicates that they have a high selectivity for aflatoxin production.

All four of the inhibitors tested are known antifungal agents. However, the aflatoxigenic fungus, *A. parasiticus*, showed high resistance against them. Atpenin A5 and siccanin strongly inhibit growth of some pathogenic fungi, such as *Trichophyton mentagrophytes* [10,11]. Siccanin strongly inhibits succinate dehydrogenase of complex II of *T. mentagrophytes*, which suggests that complex II may be the primary target for the fungicidal action of siccanin [12]. Atpenin A5 inhibits the mammalian complex II more strongly than siccanin [13], but there is no information on the inhibitory activities of atpenin A5 and siccanin on complex II of aflatoxigenic fungi and it is not clear if the inhibitory activities on the fungal complex II are parallel to their aflatoxin production inhibitory activities.

**Table 1:** Aflatoxin-production inhibitory activity of respiration inhibitors.

Classification	Target	Compound	IC_50_ (µM) *
Natural product	complex I	rotenone	13
	complex II	siccanin	13
		atpenin A5	9.7
	complex III	antimycin A	7.2
Synthetic miticide	complex I	pyridaben	0.01
		tolfenpyrad	0.18
	complex II	mepronil	23
		cyflumetofen	>300
	complex III	fluacrypyrim	0.07
		acequinocyl	1.7
		bifenazate	20
Synthetic fungicide	complex II	boscalid	<0.01
	complex III	Pyribencarb	0.43
		cyazofamid	0.70
		pyraclostrobin	0.06
		kresoxim-methyl	0.06
		azoxystrobin	0.40
		trifloxystrobin	0.90
		picoxystrobin	8.6
		metominostrobin	9.9

Note: * For production of total aflatoxin (aflatoxin B_1_ and aflatoxin G_1_).

### 2.2. Inhibitory Activities of Synthetic Pesticides with Respiration Inhibitory Activity on Aflatoxin Production

**Figure 2:**
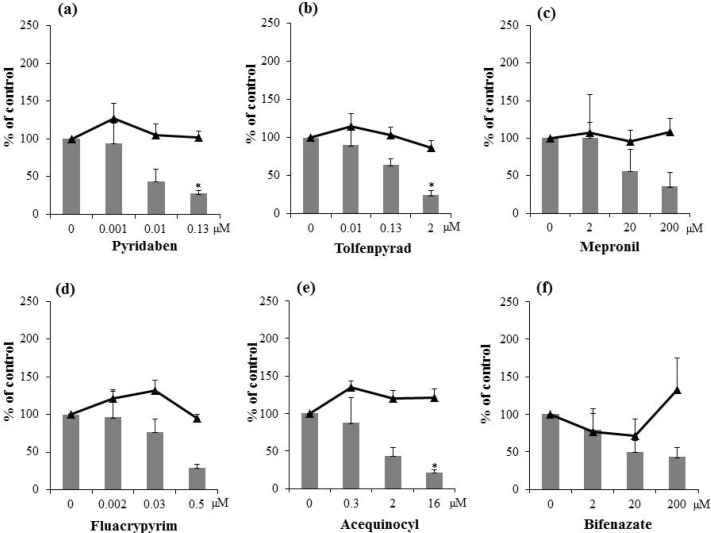
Effects of synthetic miticides, pyridaben (**a**); tolfenpyrad (**b**); mepronil (**c**); fluacrypyrim (**d**); acequinocyl (**e**); and bifenazate (**f**), on aflatoxin (total aflatoxins B_1_ and G_1_) production (gray bars) and mycelial weight (black triangles) of *A. parasiticus*. *n* = 4; * *p* < 0.05, *vs.* control.

**Figure 3:**
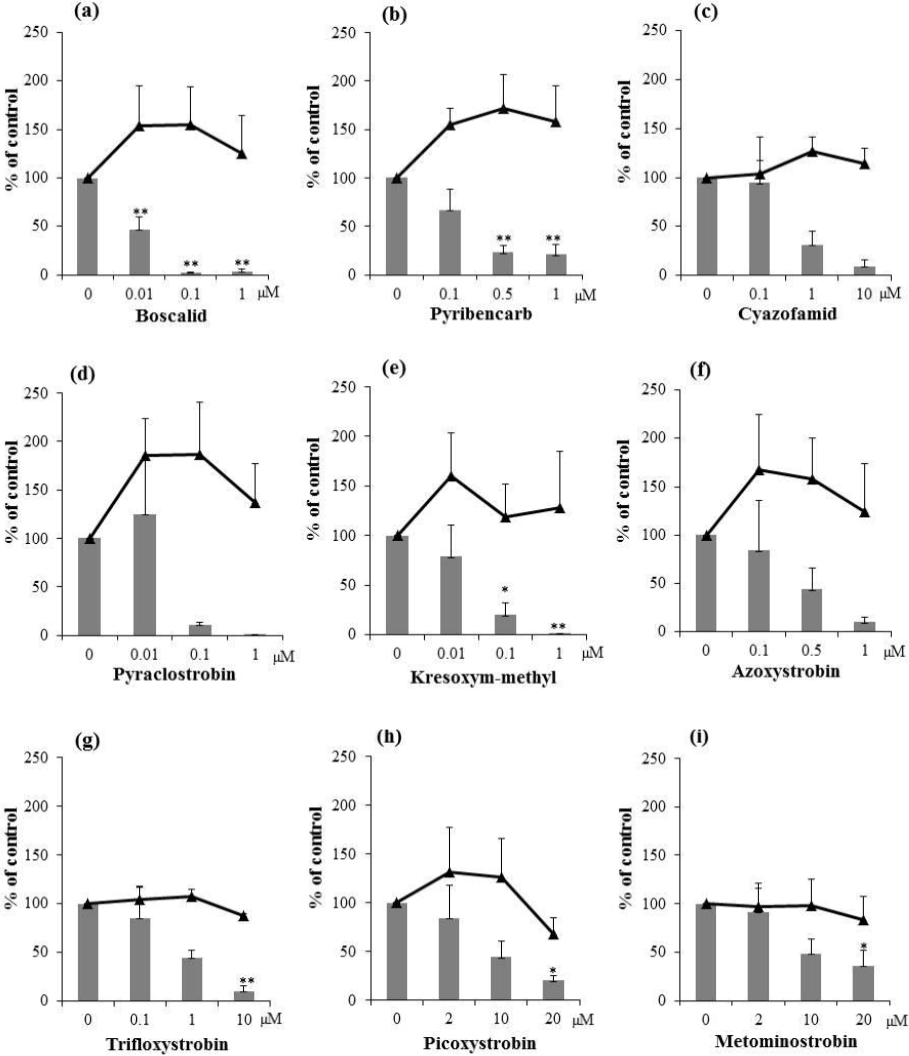
Effects of synthetic fungicides, boscalid (**a**); pyribencarb (**b**); cyazofamid (**c**); pyraclostrobin (**d**); kresoxym-methyl (**e**); azoxystrobin (**f**); trifloxystrobin (**g**); picoxystrobin (**h**); and metominostrobin (**i**), on aflatoxin (total aflatoxins B_1_ and G_1_) production (blue bars) and mycelial weight (black triangles) of *A. parasiticus*. *n* = 4–5; ** p < 0.01; * *p* < 0.05, *vs.* control.

Aflatoxin-production inhibitory activities of seven commercially available miticides and nine fungicides with respiration inhibitory activity were examined (Figure 2 and Figure 3). The IC_50_ values obtained are listed in Table 1. Among the miticides, pyridaben and tolfenpyrad (complex I inhibitors) [14] and fluacrypyrim and acequinocyl (complex III inhibitors) demonstrated stronger inhibitory activities than the natural inhibitors. Specifically, pyridaben and fluacrypyrim had IC_50_ values less than 0.1 µM. Mepronil (a complex II inhibitor) and bifenazate (a complex III inhibitor) exhibited weak activities, and cyflumetofen (a complex II inhibitor) displayed very weak activity (Table 1).

It has been shown that cyflumetofen strongly inhibits the mitochondrial complex II of the spider mite, but it does not inhibit the mitochondrial complex II of insects, crustaceans, or mammals [15]. Although it is not clear if cyflumetofen inhibits complex II of fungus, its high selectivity for inhibiting the spider mite complex II might be related to its weak aflatoxin-production inhibitory activity. We did not observe a significant reduction of fungal mycelial weight by any of the miticides tested at the concentrations tested (Figure 2a–f). This finding indicates that some miticides, such as pyridaben and fluacrypyrim, can inhibit aflatoxin production by the aflatoxigenic fungus with high selectivity.

All fungicides tested showed strong aflatoxin-production inhibitory activity (Figure 3a–i). Among them, boscalid (a complex II inhibitor) [16] and pyribencarb, kresoxim-methyl, azoxystrobin, and pyraclostrobin (complex III inhibitors) [17] inhibited aflatoxin production strongly with IC_50_ values comparable to those of pyridaben and fluacrypyrim mentioned above (Table 1). Since none of the fungicides significantly reduced fungal mycelial weight at the concentrations tested (Figure 3), these fungicides also show high selectivity for inhibiting aflatoxin production. Salicylaldehyde was previously shown to enhance the anti-fungal activity of antimycin A and kresoxim-methyl against aflatoxigenic fungi [18], but aflatoxin-production inhibitory activities of antimycin A and kresoxim-methyl were not reported.

Overall, the current study examined inhibitory activities of 20 compounds on aflatoxin production. From the results summarized in Table 1, it is difficult to identify a correlation between the targets of the respiration inhibitors (complexes I, II, and III) and their IC_50_ values for aflatoxin-production inhibitory activity, suggesting that respiration inhibitors with a variety of targets may have a potential for inhibiting aflatoxin production. Work that investigates the mode of action of respiration inhibitors for inhibition of aflatoxin production is currently in progress.

## 3. Experimental Section

### 3.1. Strains, Chemicals, and Culture Conditions

*Aspergillus parasiticus* NRRL 2999 was used as a producer of aflatoxins B_1_ and G_1_ throughout the study [19]. Aflatoxins B_1_ and G_1_ are the main aflatoxins produced by the NRRL 2999 strain. NRRL 2999 was maintained on potato dextrose (PD) agar (Difco, MD) and subcultured monthly. A spore suspension prepared from a week-old culture at a concentration of 2.7 × 10^3^ cells/µL was used as the inoculum. The spore suspension (30 µL/well) was inoculated into PD liquid media in 24-well microplates (1 mL/well). All test compounds were dissolved in dimethyl sulfoxide and added to the wells (final concentration of dimethyl sulfoxide was 0.1% v/v). The plates were incubated undisturbed at 27.5 °C for three days.

Siccanin and atpenin A are fungal metabolites obtained from the natural products library of the Kitasato Institute for Life Sciences. Rotenone and antimycin A were purchased from MP Biomedicals, LLC, Illkirch, France and Sigma-Aldrich, St. Louis, MO, USA, respectively. Cyflumetofen was gifted from Dr. Ikemi of Otsuka Chemical Co., Ltd. Pesticides except for cyflumetofen were purchased from Wako Pure Chemical Industries, Ltd., Osaka, Japan.

### 3.2. Analysis of Aflatoxin

After three days of incubation, the NRRL 2999 culture broth of each well was centrifuged to obtain the mycelia and the culture supernatant. The mycelia were washed with 1 mL of distilled water two times and collected by centrifugation in a 1.5 mL microtube. After freeze-drying the mycelia, the mycelial weight was calculated by subtracting the weight of an empty 1.5 mL microtube from the total weight.

Aflatoxins B_1_ and G_1_ in the culture supernatant were analyzed in the following manner. The supernatant (0.7 mL) was extracted with 200 µL of chloroform, and the chloroform solution was distilled off by air-drying. The remaining residue was dissolved in 0.1 mL of a 90% aqueous acetonitrile solution. The dissolved mixture was subjected to reverse-phase HPLC on a 250 mm × 4.6 mm i.d. Capcell pak C_18_ UG120 column by an isocratic elution of acetonitrile:methanol:water (10:30:60) over 20 min at a flow rate of 1.0 mL with detection at 365 nm to quantify aflatoxins B_1_ (retention time: 8.3 min) and G_1_ (retention time: 11.1 min).

### 3.3. Data Analysis

The NRRL 2999 strain was cultured in PD liquid medium in a well of microplate, with or without a sample, at 27.5 °C for three days. Amounts of aflatoxins B_1_ and G_1_ in the culture supernatant and mycelial weight were measured according to the methods mentioned above. This experiment was repeated four or five times (*n* = 4–5). The aflatoxin amounts and mycelial weight of each well were normalized to those of control well. The normalized values were used for statistical analysis. Data are presented as the mean ± SE. Differences between groups were assessed by Dunnett’s test.

## 4. Conclusions

The results obtained in this study indicate that respiration inhibitors can control aflatoxin production, and a microbe that produces a respiration inhibitor, such as antimycin A, has the potential to be a bio-control agent. There are a number of commonly used synthetic pesticides that exhibit respiration inhibitory activity, including those used in this study. This study suggests that those pesticides may be also useful as aflatoxin control agents.
